# Impact of the Lung Microbiota on Development and Progression of Lung Cancer

**DOI:** 10.3390/cancers16193342

**Published:** 2024-09-29

**Authors:** Amine Belaid, Barnabé Roméo, Guylène Rignol, Jonathan Benzaquen, Tanguy Audoin, Valérie Vouret-Craviari, Patrick Brest, Raphaëlle Varraso, Martin von Bergen, Charles Hugo Marquette, Sylvie Leroy, Baharia Mograbi, Paul Hofman

**Affiliations:** 1 Université Côte d’Azur, Institute for Research on Ageing and Cancer, Nice (IRCAN), Institut Hospitalo Universitaire (IHU) RespirERA, Fédérations Hospitalo-Universitaires (FHU) OncoAge, Centre Antoine Lacassagne, Centre national de la recherche scientifique (CNRS), Institut national de la santé et de la recherche médicale (INSERM), 06107 Nice, France; amine.belaid@univ-cotedazur.fr (A.B.); barnabe.romeo@univ-cotedazur.fr (B.R.); rignol.g@chu-nice.fr (G.R.); benzaquen.j@chu-nice.fr (J.B.); tanguy.audoin@etu.univ-cotedazur.fr (T.A.); valerie.vouret@unice.fr (V.V.-C.); patrick.brest@univ-cotedazur.fr (P.B.); marquette.c@chu-nice.fr (C.H.M.); leroy.s2@chu-nice.fr (S.L.); hofman.p@chu-nice.fr (P.H.); 2 Laboratory of Clinical and Experimental Pathology (LPCE), Biobank (BB-0033-00025), Centre Hospitalier Universitaire (CHU) de Nice, FHU OncoAge, IHU RespirERA, 06000 Nice, France; 3 Centre Hospitalier Universitaire (CHU) de Nice, Department of Pulmonary Medicine and Thoracic Oncology, FHU OncoAge, IHU RespirERA, 06000 Nice, France; 4 Université Paris-Saclay, Équipe d’Épidémiologie Respiratoire Intégrative, CESP, INSERM, 94800 Villejuif, France; raphaelle.varraso@inserm.fr; 5 Helmholtz Centre for Environmental Research GmbH—UFZ, Department of Molecular Systems Biology, Institute of Biochemistry, Faculty of Life Sciences, University of Leipzig, 04109 Leipzig, Germany; martin.vonbergen@ufz.de

**Keywords:** lung cancer, microbiota, air pollutant, exposome, diet, commensal, immune checkpoint inhibitors, antibiotics, probiotics, predictive biomarker, tumor microbiota

## Abstract

**Simple Summary:**

Recent research has helped us understand more about the role of microbes in the health and disease of the lungs. The detection of microbes and microbial products in sputum may improve early lung cancer diagnosis. The monitoring of the microbiome of the lungs over time may help predict the response to and side effects of treatment. However, studies have not yet examined how diet and air pollution affect the lung microbiome and how it might be linked to the development and progression of lung cancer. By examining the lung microbiome, dietary patterns, and air pollutants, we hope to prevent and manage lung cancer in the future.

**Abstract:**

The past several years have provided a more profound understanding of the role of microbial species in the lung. The respiratory tract is a delicate ecosystem of bacteria, fungi, parasites, and viruses. Detecting microbial DNA, pathogen-associated molecular patterns (PAMPs), and metabolites in sputum is poised to revolutionize the early diagnosis of lung cancer. The longitudinal monitoring of the lung microbiome holds the potential to predict treatment response and side effects, enabling more personalized and effective treatment options. However, most studies into the lung microbiota have been observational and have not adequately considered the impact of dietary intake and air pollutants. This gap makes it challenging to establish a direct causal relationship between environmental exposure, changes in the composition of the microbiota, lung carcinogenesis, and tumor progression. A holistic understanding of the lung microbiota that considers both diet and air pollutants may pave the way to improved prevention and management strategies for lung cancer.

## 1. Introduction

Lung cancer claims 2 million lives every year, accounting for almost a quarter of all cancer deaths—more than any other cancer [1]. While the lungs were once considered sterile, they are now recognized as dynamic ecosystems composed of viruses, fungi, parasites, and bacteria [2,3,4]. The lung microbiota results from a delicate balance between immigration (via inhalation, micro-aspiration, dispersion from the oropharyngeal mucosa) and elimination (via the mucociliary elevator and coughing, immune selection, or due to local abiotic factors, competition between microorganisms). The composition and abundance of the lung microbiota are influenced by multiple factors, including diet, environment, immune status, and genetic predisposition [5]. Numerous studies have undeniably linked the lung microbiota to respiratory health and changes in its composition to the development of lung cancer, among other lung diseases [6,7]. As we delve deeper into the tumor, bacteria of the lungs emerge as unanticipated players that shape the immune tolerance of the lungs and the immune response of the tumors. Drawing from compelling preclinical and clinical studies, interventions such as probiotics, prebiotics, and fecal microbiota transplantation (FMT) have improved the efficacy of immune checkpoint inhibitors (ICIs) while reducing adverse immune reactions via targeted manipulation of the microbiota [8]. This review explores how mining the lung commensals and tumor microbiomes holds promise to identify those patients who will benefit from immunotherapy accurately and to guide the choice of new ICI combinations to maximize the efficacy of ICIs.

## 2. Approaches for Studying the Lung Microbiome: Techniques and Challenges

Recent advances in sequencing and metagenomics have demonstrated the presence of a broad biodiversity in the microbiome of the respiratory tract [9,10,11]. The specific microbiomes, unattainable through conventional culture-based methods, open the way to new promising therapeutic and diagnostic avenues. Below, we will briefly discuss the basic principles of the techniques of studying the lung microbiome, emphasizing the challenges and limitations.

### Inclusion and Exclusion Criteria for Lung Microbiota Studies

Carefully designed inclusion criteria help define a study population that accurately represents the research question. Studies often include adults (typically aged 18 to 65) with specific lung conditions such as asthma, COPD, cystic fibrosis (CF), or lung cancer. The focus on these conditions aims to explore alterations in microbiota associated with chronic lung diseases. Participants should be in a stable phase of their disease without recent exacerbations for these chronic conditions [12]. Stability ensures that the microbiota profile reflects the baseline condition of the lung disease rather than transient changes due to acute illness. Informed consent is a mandatory requirement for participation.

Exclusion criteria are designed to reduce confounding variables and protect participant health. Common exclusion criteria for lung microbiota studies include recent antibiotic use. Participants who have used antibiotics within three months before the study are excluded because antibiotics can significantly alter the composition of lung microbiota, leading to misleading results [6]. Participants with recent respiratory infections, such as the common cold, influenza, or pneumonia, are excluded because these infections can temporarily alter the lung microbiota [13]. Individuals with severe comorbidities, such as uncontrolled diabetes, cardiovascular disease, or immune disorders, are also excluded, as these conditions could affect lung microbiota composition and confound study outcomes [14]. Lastly, pregnant individuals are often excluded due to hormonal changes, immune alterations, and potential risks to both the mother and fetus. Pregnancy can significantly influence microbiota composition, making it challenging to isolate the effects of lung conditions alone [14].

By adhering to these criteria, we can enhance our understanding of the complex interactions between lung microbiota and respiratory health.

*Sample collection*. Sputum and bronchoalveolar lavage fluid (BALF) are common approaches to lung microbiome sampling but are associated with distinct limitations. Sputum is noninvasive, but it contains a mix of the microbiota of the upper and lower respiratory tracts [15]. This mixture of microbial communities complicates efforts to accurately identify the unique microbial signature of the lungs. In contrast, BALF is less influenced by oral contamination than sputum but requires an invasive procedure [16,17]. Still, the microbial composition can be inconsistent when comparing the whole BALF to a host cell-depleted BALF. This inconsistency is likely due to the removal of cell-associated bacteria during the depletion process [18].

*Analytic approaches*. The most widely used approach involves PCR amplification and sequencing of the 16S rRNA gene, a small and highly conserved locus of the bacterial genome. When a respiratory sample is sequenced, thousands of short sequences are produced and aligned on taxonomic databases. This cost-effective method helps profile the bacterial communities at the genus and species levels in a sample [8]. However, because it focuses on conserved 16S rRNA, it cannot distinguish between closely related bacterial strains.

**Figure 1:**
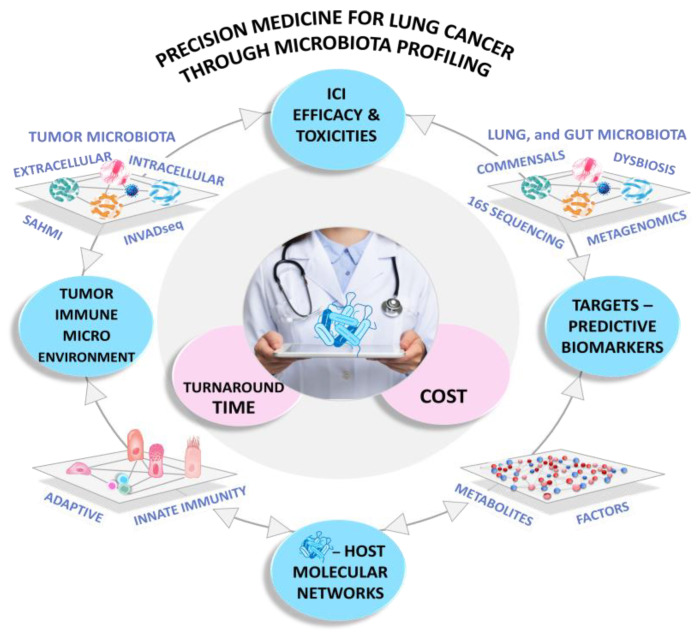
Challenges in Precision Medicine for Lung Cancer through Microbiota Profiling. Metagenomic analyses help identify specific bacterial profiles associated with the efficacy of immune checkpoint inhibitors (ICIs) or adverse effects (irAEs). Techniques like metagenomics, metabolomics, and spatial profiling offer insights into the complex molecular networks (including metabolites, antigens, inflammatory mediators, and proteases) that influence the interplay between the microbiota and systemic or tumor immunity. Recent advancements, such as INVADEseq and the SAHMI (Single-Cell Analysis of Host-Microbiome Interactions) pipeline, have demonstrated bacterial colonization within tumors and its impact on immune evasion and tumor cell migration. These findings could reveal novel targets and diagnostic biomarkers for immunotherapy. However, considering these technologies’ associated costs and turnaround times is essential before clinical implementation.

Shotgun metagenomics simultaneously captures all genetic material in a sample, from humans to bacteria, viruses, and fungi, allowing highly accurate taxonomic classification and comprehensive identification of the microbiome. One limitation is that the DNA of different species may be differentially extracted. Compared with 16S sequencing, metagenomics requires deeper sequencing and more computational analysis [8]. Despite their efficiency, it should be emphasized that both 16S rRNA sequencing and shotgun metagenomics provide snapshots that capture both living and dead bacteria [19]. In contrast, metatranscriptome analysis sequences the RNA transcripts present in a sample. This method focuses on the genes that are being transcribed, therefore identifying the living bacteria at the time of sampling. Thus, the metatranscriptome offers, with the bacterial metabolome, a more dynamic view of the microbiome’s functional state [19]. However, it is crucial to consider the cost and challenges (long turnaround time, artificial intelligence analysis, metabolomic and bioinformatic expertise) associated with this microbiome analysis before applying it to a clinical setting (Figure 1).

## 3. The Balance of the Lung Microbiome in Health and Disease

*Eubiosis*. A healthy lung microbiome is established in the first months of life and is a transient ecosystem mainly populated by oral bacteria. Firmicutes, Bacteroidetes, and Proteobacteria are the dominant species, and *Prevotella*, *Veillonella*, and *Streptococcus* are the three most common genera [2,20,21]. These bacteria coexist harmoniously within the lung in a state called eubiosis without causing harm. The migration of commensals from the oral and tracheal cavities to the lungs occurs through passive inhalation and is followed by their rapid elimination via continuous airflow and ciliary movement. Moreover, the mucus offers limited nutrients for bacterial growth, and the robust immune system (macrophages, neutrophils, antimicrobial peptides, and adaptive immune cells) targets and neutralizes any potential colonization. Collectively, these host defenses keep the lung microbiome at low biomass, constantly inhaled, killed, and replaced [5].

*Dysbiosis*. Any shifts in the quantity and variety/composition of lung bacteria, i.e., dysbiosis, are long-lasting and increase the host’s susceptibility to infection, chronic inflammation, allergies, and development of various lung diseases [22,23]. Asthma and chronic obstructive pulmonary disease (COPD) show altered lung microbiomes. In these conditions, pathogenic Proteobacteria levels increase, the genera *Haemophilus*, *Moraxella*, *Streptococcus*, *Staphylococcus*, and *Actinomyces* become more prominent, while the commensal bacteria *Prevotella* and *Veillonella* decrease [6]. The lung microbiota holds significant potential as both a diagnostic and prognostic biomarker for various broncho and pulmonary diseases [24]. Understanding these microbial shifts is pivotal to developing targeted therapies and maintaining respiratory health (Table 1).

## 4. Role of Lung Microbiota in Modulating Immune Surveillance and Tolerance: Implications for Respiratory Health

Despite its low microbial density, the lung microbiota is critical in preserving lung health. This is mainly due to the commensals that prevent pathogen colonization by competing for space and nutrients. In addition, they help prime the immune system to recognize and respond to infections through the continuous low-level stimulation of the innate immune system.

Alveolar macrophages, the primary resident immune cells in the lungs, continuously patrol the lungs, phagocytose pathogens, and present antigens to the T lymphocytes. These macrophages have pattern recognition receptors (PRRs) on their surface, which detect microbial-associated molecular patterns (MAMPs). Upon PRR stimulation, alveolar macrophages secrete cytokines such as IL-1B and IL-23, essential for differentiating TH17 cells. TH17 cells are a subset of T helper cells that rapidly recruit neutrophils to sites of infection via IL-17 [25,26].

Under healthy conditions, persistent PRR stimulation by the airway microbiota can promote the differentiation of M2 macrophage [27]. These M2 macrophages and the regulatory T cells (Tregs) produce anti-inflammatory cytokines (IL-10 and TGF-β), which help dampen aggressive Th17 cell activity against harmless commensals. Additionally, bacterial short-chain fatty acids (SCFAs) also control the differentiation and expansion of Treg/Th17 cells [28]. This well-coordinated effort ensures that the lung remains tolerant to commensals while being ready to prepare an effective response against pathogens (graphical abstract).

Tumor Suppressor. Aside from preventing infection, lung commensals prime robust immune surveillance that detects and prevents the initiation of cancer. Antibiotic-treated mice that lack these beneficial bacteria are more prone to develop respiratory infections [29,30] and Lewis lung carcinomas [31]. This increased susceptibility converges to a defective γδ Th17 cell response, and supplementing IL-17 can reverse this impaired immune surveillance [31]. Thus, through its interaction with the host immune system, the lung microbiota protects the lungs from infection, inflammatory damage, and cancer development.

## 5. Unveiling Hidden Connections: The Lung Cancer Microbiome from Pathogenesis to Treatment Response

Since at least 2015, an increasing number of studies have demonstrated that the lung microbiota in lung cancer patients significantly differs from that of healthy individuals [32,33,34,35,36]. Their BALF, brushing, or sputum samples exhibit higher DNA levels of oral bacteria such as *Veillonella*, *Streptococcus*, *Megasphaera*, *Acidovorax*, and *Bradyrhizobium japonicum* while levels of *Staphylococcus* decrease [32,33,34,35,36]. The groundbreaking works of Nejman et al., Greathouse et al., and Wong-Rolle et al. have undoubtedly highlighted the presence of bacteria within the lung tumor microenvironment [37,38,39]. These tumor-associated microbes, often found intracellularly, can directly interact with tumor and immune cells, potentially influencing the progression of tumors and response to treatment of patients [8].

*Dysbiosis.* The lung microbiomes of tumoral and non-tumoral regions of patients with lung cancer exhibit a distinct microbiome composition characterized by a higher bacterial load and reduced α-diversity than healthy individuals [32,33,34,35,36,37,40]. This reduced diversity in the microbiota of lung tumors has been linked to poor survival, poor treatment responses, and an increased risk of recurrence [41,42], raising intriguing questions about the role of bacteria in cancer progression.

*The symbiosis between tumor cells and bacteria*. Recent studies using cutting-edge methods like single-cell RNA sequencing and spatial profiling have captured bacterial species within lung tumors [37,38,39]. It is now recognized that the bacteria residing within the tumor cell cytosol are viable [43,44,45,46]. The tumor cells provide a protective niche that bacteria can invade, safe from immune attack. In exchange, bacteria of lung tumors confer multiple growth advantages to tumors rather than being merely passive inhabitants (graphical abstract).

*The microbiota: The connection between chronic inflammation and cancer initiation*. While *Helicobacter pylori* is now definitively linked to the development of gastric cancer, the specific bacteria within the lung microbiome that may directly cause lung cancer have not yet been identified. Instead, recent research has shed light on the pivotal role of lung dysbiosis in the development of lung cancer. Jin et al. discovered that removing lung commensal bacteria in mice suppresses lung cancer, even after mutation in the *KRAS* and *TP53* genes [35]. In contrast, installing lung bacteria from advanced lung cancer promotes inflammation and progression of lung cancer [35]. Rather than directly causing oncogenesis, lung dysbiosis was found to shape the tumor microenvironment. The critical steps in the chain reaction include chronic PPR activation by PAMP, the expansion of IL17-producing γδ T cells (Th17), and neutrophil-infiltrated inflammation, which favors proliferation of lung adenocarcinoma cells [47,48]. The inflammatory microenvironment created by lung dysbiosis has been correlated with the activation of several oncogenic signaling pathways (MAPK/ERK, PI3K, JAK-STAT, NFκB, and Wnt/β-catenin) [36,39,41,49]. Understanding these mechanisms may provide new insight into preventing and treating lung cancer by targeting the lung microbiota.

*Immunosuppression.* Like any ecosystem with predators and prey, the microbiome protects the tumor cells from immune attack of the hosts. The bacteria or fungi within the tumor release pathogen-associated molecular patterns (PAMPs), such as flagellin, lipopolysaccharides, and β-glucan, which activate various signaling pathways (MAPK, JAK-STAT, NFκB). These pathways converge on the expression of chemokines (IL-1β, IL-6, IL-8, IL-17, TNF) and the recruitment of immunosuppressive myeloid-derived suppressor cells, regulatory T cells, and neutrophils into the tumor bed [41,50]. Within this immune sanctuary, the immune checkpoints PD-1 are up-regulated on T cells, and CD8+ T cells are excluded from the bacteria-rich areas [41,51]. According to Susan Bulman’s proposal, cancer and bacteria cooperate in immune escape strategies, leading to mutual symbiosis and survival [46].

*The Immunotolerance induced by the lung microbiota is a double-edged sword.* On the positive side, it helps to maintain a balanced immune response and prevent unnecessary inflammation that could damage the lungs. However, on the negative side, it can inadvertently protect lung cancer cells from the antitumor immune response. The study by Zagorulya et al. provides valuable insight into the mechanisms by which lung cancer exploits this microbiota-induced immune tolerance to evade immune surveillance and the effects of immunotherapy (graphical abstract) [52]. Within the lymph nodes that drain the lung carcinoma, the interaction between immature T cells, dendritic cells, lung commensals, and Treg cells creates an environment in which CD8+ T cells are unable to mature and fully exert their cytotoxic effects on tumor cells. This compromises the effectiveness of immune checkpoint inhibitors [52]. The recent research by Battaglia et al. (2024) highlights the role of *Fusobacterium nucleatum*, a Gram-negative anaerobic commensal, in creating a tolerogenic environment and contributing to ICI resistance in non-small cell lung cancer (NSCLC) [53]. Fusobacterium-high tumors exhibit lower cytotoxic, IFNG, and MHC gene expression signatures, compromising the immune response against the tumor [53].

Further investigations into the role of *F. nucleatum* should lead to new adjunct strategies to improve the outcomes of immunotherapy, such as using probiotics or targeted antibiotics to modulate the lung microbiota. This approach could potentially enhance the ICI effectiveness and improve the survival rate of patients with lung cancer.

*Source of tumor-specific antigens: Insights from the tumor microbiota.* While the above evidence suggests an immunosuppressive role of the tumor microbiota, it may also play a critical role in training the immune system to target tumors by presenting bacterial antigens. Several bacteria (*Actinomyces*, *Bacteroidetes*, and *Prevotella*) within the cytosol of melanomas can be targeted for degradation, leading to antigen processing and presentation to HLA molecules [54]. These bacterial peptides act as tumor neoantigens, significantly activating T cells in vitro [54]. Consistently, these genera produce immunogenic peptides and have recently been demonstrated to recurrently drop in ICI-responsive patients [53].

These discoveries, viewed through the “microbe lens”, offer a new perspective into the anti-cancer immune response. It has long been postulated that bacteria may produce epitopes mimicking tumor neoantigens, potentially educating lymphocytes to attack tumor cells through antigen mimicry [55]. We now appreciate that the intracellular bacteria are the source of these tumor neoantigens. Thus, the T cells may target the tumor cells as bacteria-infected cells. Given their tumor-specific nature and shared characteristics among patients, these bacterial peptides hold potential as tumor vaccines [56].

*Cancer progression and metastasis.* The higher abundance of *Veillonella parvula* and *Acidovorax temperans* in the lungs accelerates tumor development by infiltrating immunosuppressive and proinflammatory cells into the tumor microenvironment. This lower airway dysbiotic signature is present in patients with advanced/metastatic (stage IIIB–IV) NSCLC and is associated with poor clinical prognosis [41,48]. Other intracellular bacteria like *B. fragilis*, *Staphylococcus*, *Lactobacillus*, and *Streptococcus* persist in metastatic colorectal and breast cancer cells [44,57,58]. These bacteria drive tumor cells to undergo epithelial-mesenchymal transition and inhibit RhoA-ROCK [44]. This mechanism enables clustered circulating tumor cells (CTC) to withstand mechanical stress in blood vessels [44]. Experiments with germ-free mice have shown that inoculating bacteria into tumors can sufficiently promote lung metastases [44]. Therefore, deciphering the role of the microbiota in the dissemination of tumor cells is essential to developing effective treatments for metastasis, which is the leading cause of cancer-related deaths.

## 6. Air Pollution and the Risk of Lung Cancer: Is the Lung Microbiome Our Defense or Vulnerability?

Cigarette smoke (CS) and air pollution are major contributors to lung cancer [59,60]. Around half of smokers will develop COPD and cancers, with more than 80% of lung cancers being linked to CS exposure [59,60]. However, the growing incidence of lung cancer among non-smokers highlights the critical impact of air pollution, especially particulate matter [61]. Particulate Matter (PM2.5 and PM10) can penetrate deeply into the lungs, reaching the alveoli and then the bloodstream, and has been classified as a carcinogen. In 2020, this alarming trend prompted the World Health Organization (WHO) to lower the global air quality standards [62]. At the dawn of the 21st century, we also face the critical challenge of global warming. Rising temperatures can foster the growth of specific bacteria and fungi. At the same time, changes in air pollution and particulate matter due to climate change can influence the types of microbes inhaled and deposited in the respiratory tract. Together, these global changes—warming and pollution—can significantly disrupt the balance of microbial communities in the lungs, potentially increasing the risk of infection and lung cancer and exacerbating conditions such as asthma and COPD [63]. Currently, 90% of the global population is breathing polluted air, and 10 million people die every year from the acute and cumulative effects of air pollution, totaling 100 million a decade. These figures make air pollution a more significant threat to human mortality than the COVID-19 pandemic over the past four years [2,22].

Despite these alarming statistics, the specific pollutants and impaired physiological mechanisms underlying lung inflammation and carcinogenesis remain poorly understood. Recent evidence reveals that CS and air pollutant exposures target the lung microbiome. The study of Swanton et al. emphasizes the significant role of air pollution in lung carcinogenesis, even among non-smokers [59]. It suggests that environmental pollutants can influence the lung microbiome and contribute to mutations in the epidermal growth factor receptor (EGFR), which is a key driver in certain lung cancers [59]. This research underscores the need for a deeper understanding of how air pollutants drive these genetic changes and calls for tailored prevention strategies to mitigate these risks.

*Our defense.* As discussed earlier, a well-balanced lung microbiome in healthy lungs helps mitigate excessive inflammatory responses, making healthy individuals less susceptible to pollutants.

*Our vulnerability.* Persistent exposure to CS or pollutants disrupts the lung and gut microbiomes, decreasing α-diversity [15,64]. Exposure to CS fosters the growth of Firmicutes and a decline in anti-inflammatory Bacteroidetes, thus compromising the protective functions of the microbiome and increasing susceptibility to pollutant toxicity [39,64]. Specific bacteria such as *Staphylococcus haemolyticus*, *Acidovorax*, and *Haemophilus influenzae* are enriched in smokers with lung cancer [32,34,37]. This NSCLC microbiota harbors metabolic pathways that convert chemicals in CS (such as nicotine, anthranilate, toluene, and phenol) into more toxic and carcinogenic compounds, potentially increasing the risk of cancer [38].

Given that air pollution is a significant threat to global health, we claim that understanding how CS and air pollutants disrupt the lung microbiome and contribute to lung diseases can improve public health strategies and interventions. We hope, in the near future, that restoring a healthy lung microbiome through probiotics, prebiotics, and other interventions could mitigate the adverse effects of pollutants and cigarette smoke.

## 7. The Gut–Lung Axis (GLA) Is an Ally in the Effectiveness of Immune Checkpoint Inhibitors

The latest therapeutics for lung cancer increase the interplay between the host immune system and cancer cells [65]. ICIs are monoclonal antibodies that block immune system inhibitors, empowering immune cells to identify and combat cancer cells. This breakthrough has led to sustained remission for NSCLC patients. However, the therapy remains costly and is only effective in 20% of patients.

**Figure 2:**
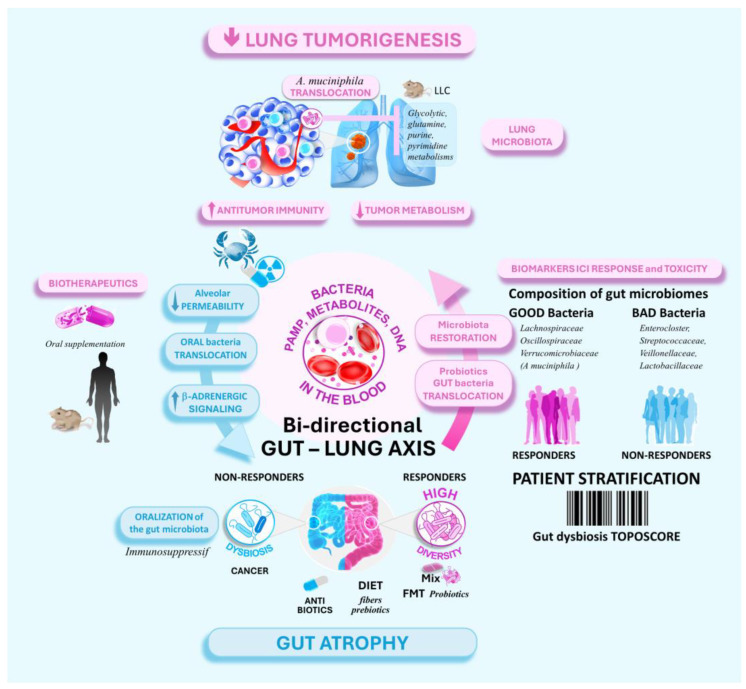
The gut–lung axis (GLA) plays a crucial role in the effectiveness of ICIs for NSCLC patients. It links the gut and lung microbiomes and significantly influences the development of lung cancer and response to immunotherapies. The composition of the gut microbiome can categorize NSCLC patients into responders and non-responders to immunotherapy. Typically, responders have a more diverse gut microbiome characterized by a higher level of beneficial bacteria. The gut bacteria can directly impact the host’s immune cells and even travel through the bloodstream to activate immune cells in the lungs. Additionally, gut bacteria can colonize lung tumors and suppress tumor growth by modifying the tumor microbiome and reprogramming tumor metabolism. Interventions like fecal microbiota transplantation (FMT) and probiotics offer promising strategies to modulate the gut–lung axis, potentially improving the therapeutic outcomes of cancer patients. ↑ increased, ↓ decreased.

Several groundbreaking studies have established an intricate link between the gut and lung microbiomes, termed the gut–lung axis (Figure 2). In this bidirectional relationship, the gut microbiome can affect the immune response in the lung and, therefore, the response of lung cancer to immunotherapies [66,67,68]. Additionally, lung cancer and its treatments can induce gut dysbiosis [69]. Many immunosuppressive microbes from the oral cavity can transit to the small intestine, where they cause beta-adrenergic-dependent stress ileopathy, gut dysbiosis, and gut permeability, favoring the systemic spread of immunosuppressive metabolites and components [69,70].

Subsequent studies by Routy et al., Gopalakrishnan et al., and Matson et al. demonstrated the ability to categorize patients into responders and non-responders to immunotherapy based on the composition of their gut microbiome [71,72,73,74]. In another study, Derosa et al. applied a species network clustering approach to narrow down the species that can predict the overall survival of NSCLC. The gut microbiome of non-responders was characterized by reduced α-diversity and the overgrowth of 37 immunosuppressive bacterial species. These species belong to the *Enterocloster*, *Streptococcaceae*, V*eillonellaceae*, and *Lactobacillaceae* families and induce resistance to PD-1 blockade [70].

In contrast, responders generally exhibited higher gut microbiome α-diversity, associated with increased infiltration of CD8+ T cells into tumors (Figure 2) [67,71,73,74,75,76]. A total of 45 beneficial species of gut bacteria have been identified, such as *Bifidobacterium pseudocatenulatum*, *Roseburia* spp, and *Akkermansia muciniphila*, which may be linked to the clinical benefit in NSCLC patients receiving immunotherapy [77,78]. The abundance of these species-interacting groups (SIGs) may serve as promising predictive biomarkers to stratify patients for anti-PD-(L)1 immunotherapy [70].

The precise mechanisms by which the gut commensals influence distant lung tumorigenesis, and the ICI response are unclear. Several hypotheses have been proposed but are not mutually exclusive (see Figure 2). The gut bacteria may educate the host immune cells through direct interaction. Alternatively, the microbes may release metabolites and products that can enter the bloodstream, mobilizing the immune cells in the lungs [79,80]. Additionally, bacteria can colonize lung tumors and suppress tumor growth by altering the tumor microbiome and reprogramming tumor metabolism, as recently reported for the gut *Akkermansia muciniphila* in Lewis lung cancer mouse models (LLC) [81]

*A paradigm shift: Microbiota-derived metabolites.* Two metabolites of the gut microbiome, L-arginine [82,83,84] and short-chain fatty acids, emerge to control the effectiveness of ICI for NSCLC patients. NSCLC patients who respond to ICI have higher plasma and feces levels of SCFAs, as well as L-arginine, than non-responders. Specifically, L-arginine enhances the cytotoxic activity of CD8+ T cells. Likewise, the SFCA butyrate enhances the anti-PD-1 therapy by increasing histone 3 lysine 27 acetylation (H3K27ac) at the promoters of the *PDCD1* and *CD28* genes in CD8+ T cells, thus enhancing their expression. Furthermore, supplementing with butyrate increases the cytotoxic activity of CD8+ T cells by modulating the T-cell receptor signaling pathway [85]. These compelling findings underscore the potential of the gut microbiota and its SCFAs as predictive biomarkers for ICI efficacy. They also favor the use of SCFAs as therapeutic adjuvants to improve the outcomes of NSCLC patients on ICIs.

## 8. Exploring the Lung Microbiome in Clinical Settings

### 8.1. Predictive Biomarkers

Less than five years ago, lung tumors were thought to be sterile, but now evidence shows that they are undoubtedly colonized by bacteria. Such polymorphic microbiomes are consistently implicated in host health and tumor development. In 2022, Hanahan officially accredited the tumor microbiome as a new hallmark of cancer [86]. However, most studies into the microbiota of lung tumors have relied on bronchoalveolar lavage (BAL), bronchoscopic brushing, or sputum samples [32,33,34,36,87,88,89,90]. Only a few studies have directly characterized the microbiome in tissues of lung tumors [38,39,40,91].

However, translating the microbiome data into a “universal” microbial biomarker remains challenging in daily practice in order to separate healthy from cancerous lungs [8]. The variability of microbial profiles due to different sampling methods (sputum, BALF, bronchial brush, tumor biopsies) makes this process complicated. Additionally, the dynamic turnover of the lung microbiomes within and across individuals, plus the high risk of contamination, limits the identification of specific lung tumor—and commensal bacterial species [92]. Moreover, each detection technology faces limitations. The traditional culture of microorganisms may not be sensitive enough to capture a low microbial load or non-culturable microorganisms of the lungs. While 16S rRNA gene sequencing can identify a broad range of bacteria in a single run, it does not detect fungi and viruses. Advanced techniques like next-generation sequencing (NGS) and metagenomics offer a better sensitivity but they struggle to accurately distinguish commensals and tumor-associated microbes. Consequently, only a small subset of lung tumor—and commensal bacteria has been identified [6,93].

As a result, most studies have focused on the gut–lung axis in lung cancer development and treatment response [94]. Different gut microbiota signatures have been associated with an increase in antitumor immunity and a PD-1 blockade response [71,72,73,74]. A step forward, Derosa et al. have successfully developed a 21-bacteria qPCR-diagnostic score called TOPOSCORE [70]. This score can predict gut dysbiosis and overall survival in patients with NSCLC, melanoma, and colorectal cancer treated with immunotherapy, bringing us closer to achieving microbiota-based precision medicine [70]. Alternatively, distinct biomarkers in serum could represent indicators of gut dysbiosis. Indeed, Fidelle et al. found that soluble MAdCAM-1 is a surrogate marker of over-representation of the *Enterocloster* genus, found in overt dysbiosis such as those induced by antibiotics or chronic inflammatory processes (including advanced cancers) [95].

While the gut microbiome provides insight into the overall host immune status, it may not capture the specific microbial dynamics within lung tumors. Increasing evidence suggests that bacteria within tumors may migrate from the local lower airway [26]. This emphasizes the importance of studying the lung microbiome and the microbiomes of lung tumors directly rather than relying on analysis of the gut microbiome. Several bacterial species, such as *Veillonella*, *Megasphaera*, *Bradyrhizobium japonicum*, *Streptococcus*, and *Acidovorax*, can help to distinguish patients with lung cancer from healthy controls and those with benign conditions [32,34,36,88,89,91,96]. Similarly, different lung cancer subtypes (adenocarcinoma (*Capnocytophaga*), squamous cell carcinoma (*Acidovorax* and *Veillonella*), and small cell carcinoma have distinct microbiota profiles [34,39,87,89]). Moreover, changes in the lung microbiome that can be detected in the early stages of cancer [34,40] may predict the risk of recurrence (*Acinetobacter johnsonii Acinetobacter lwoffii* and *Roseburia* [42,97]) or are associated with specific mutations (*Acidovorax*, and *TP53* [37]). This makes the lung microbiota a highly valuable biomarker for diagnosis and prognosis, offering significant potential to enhance the management of lung cancer.

Since obtaining specimens of lung tissue from patients with advanced lung cancer is challenging, using microbiota profiles from sputum, BALF, saliva, and plasma associated with lung cancer is a better alternative [32,34,42,87,88,89,98]. Identifying a microbial biomarker from a noninvasive liquid biopsy could also be promising for early cancer diagnosis, prognosis, and monitoring of the therapeutic efficacy. However, to fully integrate such lung microbiota into clinical practice, we advocate a large-scale collaborative effort to identify specific bacterial species within lung tumors and validate their use as biomarkers and therapeutic targets.

### 8.2. Hope or Hype of Turning the Microbiota into Drugs for ICI Combinations

Moving forward, targeting the tumor microbiota rather than tumor cells may represent a promising strategy to sensitize “cold” tumors to immunotherapy. Since the first proof of concept was demonstrated in mice, the number of studies exploring the microbiota and immunotherapy has surged in the last five years, totaling 2000 studies, but only 200 concerned the lungs. Various microbiome-based therapeutics are being developed, ranging from “soft” prebiotics, probiotics, diet supplements, and metabolite cocktails to FMT, engineered bacteria, bacteriophages, and the antibiotics’ “bazooka” arm [99].

*Fecal microbiota transplantation* effectively treats *Clostridium difficile* infections, achieving a success rate of 95% [100]. In independent clinical reports, patients with ICI refractory melanoma or lung cancers have shown objective clinical responses by combining anti-PD-1 therapy with FMT from responders [71,101,102]. FMT has also been effective in managing refractory ICI-related colitis [103,104]. The fact that FMT facilitated changes in the gut microbiome in all recipients suggests that an “ICI-favorable” microbiota may contribute to the FMT therapeutic activity by competing and successfully persisting [101,102]. Nevertheless, FMT is not a miracle cure. It may fail when ICI resistance is independent of the gut microbiome, when the donor and recipient have an incompatible microbiota, or when it transmits pathogens or multidrug-resistant bacteria to immunocompromised cancer patients [105]. The selection of “ideal” donors is critical to the success of the ICI-FMT combination, whether they are from healthy close relatives or unrelated ICI responders. Identifying specific bacterial strains that enhance responsiveness to ICIs may lead to a safe microbiota-based precision medicine approach.

*Antibiotics* are extensively used since infections are the second leading cause of death among cancer patients [106]. The pioneering study of Fu et al. demonstrated that the systemic and tumor deliveries of antibiotics exert divergent effects on tumor progression. Administration of antibiotics in drinking water (that eliminates both gut and tumor microbiomes) led to the regression of primary tumors [44]. Conversely, intravenous administration of antibiotics (that specifically deplete tumor-associated bacteria) inhibited lung metastases without impacting the growth of the primary tumor. This emphasized that the gut microbes influence the growth of the primary tumor, while the tumor bacteria are required for metastasis. The tumor microbiota could thus become a new actionable target for metastasis in various cancer types. Looking ahead, inhalation of antibiotics or novel antibiotic nanoparticles offers a safer strategy to administer antibiotics to patients with lung cancer, as they specifically target the lung microbiome while leaving intact the gut microbiota [5,107].

*Caution about the overuse of antibiotics.* The enthusiasm for antibiotic use is tempered by the risk of cancers linked to antibiotic overuse [108,109,110]. Several retrospective studies have cautioned against the prolonged use of broad-spectrum antibiotics, as they reduce the effectiveness of ICIs in lung cancer patients [111,112]. Likewise, administering antibiotics to ICI-treated NSCLC patients can lead to severe and potentially life-threatening immune-related adverse events, requiring the immediate cessation of ICI therapy [113,114]. Given these dual effects, a nuanced approach to antibiotic use is essential, especially in cancer patients. With 50–70% of patients with lung cancer experiencing pulmonary infections, antibiotic regimens should be tailored to specifically manage infection-causing bacteria while preserving the beneficial lung and gut microbiota, considering the specific type, dosage, and administration route.

### 8.3. Decoding the Diet-Gut–Lung Microbiome Connection in the Outcomes of Lung Cancer

Several epidemiological studies support the notion that diets rich in fruits, vegetables, and fiber, such as the Mediterranean diet (MED), are associated with a reduced risk of lung cancer [115,116]. Conversely, the Western diet, with its high intake of red and processed meats, is linked to an increased risk of lung cancer, particularly in populations with high rates of smoking [117,118,119].

*Holistic diet approach Over Supplements*. Accumulating evidence suggests that a complete diet is more effective in preventing lung cancer than taking isolated nutrients in supplement form [115,120]. For example, dietary carotenoids, which are abundant in fruits and vegetables, have been associated with a reduced risk of lung cancer [121]. However, taking high-dose carotenoid supplements, particularly beta-carotene, has been linked to an increased risk of lung cancer in smokers [122,123].

Several conceptual and methodological limitations have been raised [124,125] as people consume meals composed of a complex combination of foods rather than isolated foods or nutrients. These foods contain nutrients that may interact, making it difficult to disentangle their isolated or joint effects. Various dietary scores have been developed to assess the combined impact of a person’s diet. One such example is the Mediterranean diet (MED) score, which is characterized by a high level of consumption of fruits, vegetables, nuts, seeds, olive oil, and unrefined grains, moderate intake of fish, minimal poultry, and the least possible intake of red and processed meats [126]. Recent evidence suggests that strong adherence to the MED is associated with a reduced risk of lung cancer, particularly among former smokers [115]. In addition to dietary scores, an overall diet can be evaluated using statistical techniques that summarize a person’s dietary exposure by identifying patterns in what they eat [124,125]. A recent systematic review indicates that dietary patterns characterized by a high intake of vegetables and fruits, a low intake of animal products, and anti-inflammatory products were associated with a reduced risk of lung cancer [120], highlighting the importance of a balanced diet for good health.

*Diet and Gut–Lung Axis Hypothesis*. The precise mechanism(s) underlying these diet-lung cancer associations remain largely unknown, and mechanistic studies are lacking. In addition to directly reducing inflammation and oxidative stress, emerging evidence suggests that diet is a driving factor in shaping the composition and function of the gut microbiome [127,128], which can influence host immunity by producing locally and systemically active metabolites. One overarching hypothesis might be that distinct dietary intake affects the lung microbiome through the “gut–lung axis” and, ultimately, the risk of developing lung cancer.

Accumulating evidence supports the beneficial effects of a MED diet on the diversity, composition, and functions of the gut microbiota [129,130]. Notably, dietary fiber is well known to interact directly with gut microbes [129,131,132,133]. A recent study has shown that a fiber-rich diet boosts the response of melanoma patients to PD-1 inhibitors [74]. Additional preclinical studies have demonstrated a high level of T-cell infiltration into the TME of mice receiving a high-fiber diet. One contributing mechanism concerns the higher abundance in the gut of two commensal bacteria, *Ruminococcaceae* and *Faecalibacterium prausnitzii*. These bacteria digest fiber and produce propionate, an SCFA with immunomodulatory and antitumor effects [74,134].

While the beneficial effects have been well-studied, less attention has been paid to the adverse effects of dietary intake on the gut microbiome and metabolome. Preliminary findings suggest that intake of meat (including red and processed meats) may influence the abundance of several gut microbes [129,131]. Deciphering the underlying mechanisms could pave the way to diet-based strategies in lung cancer prevention.

### 8.4. Harnessing the Promise of Probiotics

Probiotics are dietary supplements that restore the gut microbiota by supplementing beneficial bacteria or stimulating bacterial growth. Yet, two recent studies have shown that probiotics may undermine the response to immunotherapy in melanoma and pancreatic cancer, challenging their use [74,135]. For example, when two probiotics (*Bifidobacterium* or *Lactobacillus rhamnosus*) were given to mice, they developed larger melanomas, reduced T-cell infiltration, and an impaired ICI response [74]. Some of the underlying insight into the molecular mechanism comes from studies on pancreatic cancer, in which gut *Lactobacillus* was shown to reprogram the pancreatic tumor immune microenvironment by metabolizing dietary tryptophan. The bacterial tryptophan metabolites, indoles, activate the aryl hydrocarbon receptor (AHR) in tumor-associated macrophages, promoting their immune-suppressive M2 polarization [134]. In contrast, other preclinical trials have suggested that the probiotic mixture PRO2101 alleviates chemotherapy-induced dysbiosis (gemcitabine+nab-paclitaxel) and side effects in mice [135]. Administration of live butyrate-producing bacteria (*Clostridium butyricum* strain, CBM588) increased the clinical benefit of ICI therapy (Nivolumab plus ipilimumab, NCT03893422) in patients with metastatic renal cell carcinoma or NSCLC [136,137]. These conflicting results require clinical trials with large cohorts and across different tumor types to validate the safety and benefit of probiotics.

Currently, respiratory probiotics and prebiotics are not available. However, there is promising evidence that *Lactobacillus* probiotic levels are decreased in lung tumors [40], and supplementing with *Lactobacillus* enhances the tumor immune response against Lewis lung cancer [138]. Likewise, instilling a mix of human oral commensals (*Prevotella melaninogenica*, *Veillonella parvula*, and *Streptococcus mitis*) into the lower respiratory tract of mice has significantly reduced their susceptibility to respiratory infections [30]. These findings highlight the potential of microbiome-based therapies in treating lung cancer. Our current priority is to pinpoint the specific microbial species linked to decreased inflammation. This could help restore a healthy lung microbiome to prevent and treat lung cancers.

*Manipulation of the microbiota is a step closer to reality.* Recently, a breakthrough has brought us closer to achieving this. Bender et al. provided evidence that the probiotic *Lactobacillus reuteri* was not confined to the gut but also translocated to melanomas. *L reuteri* produced indole-3-aldehyde (I3A) within the tumor environment, therefore locally activating CD8+ T cells [139]. Notably, supplementation with I3A or a tryptophan-enriched diet was sufficient to enhance the efficacy of anti-PD-L1/anti-CTLA-4 immunotherapy in preclinical melanoma. This unexpected finding supports the development of novel dietary and metabolite/ICI combinations in resistant cancer patients, bypassing the inconsistencies of probiotics and FMT.

Before introducing probiotics for clinical use, long-term investigations into the safety of different host diets and treatments, particularly genetically engineered probiotics, need to be conducted. Moreover, it is worth noting that indoles signal through the AhR in tumor-associated macrophages but reduce the efficacy of immunotherapy in pancreatic cancer [134]. This implies that the effects of probiotics and their metabolites depend on the cancer type, highlighting the necessity for further research into the role of microbial metabolites in different cancer models.

## 9. Conclusions

The field of immuno-oncology has never been as promising as it is presently. The revolution in metagenomics and spatial profiling has highlighted the microbiota as an unexpected game changer in the efficacy of ICIs. Both preclinical and clinical studies have demonstrated that manipulating the microbiota can effectively “warm” a cold tumor microenvironment, making it more responsive to ICIs. However, research on the lung microbiota is still in its infancy. There are inconsistencies in the microbiota profiles of lungs, mainly due to the sampling methods and technical approaches. Since many bacteria species of lungs and tumors are difficult to culture, most current findings are only correlations that do not prove a causal effect of the microbiome. To fully harness the potential of immunotherapy for lung cancer, we urgently need a deeper understanding of beneficial bacterial species and their impact on the tumor immune response. The question remains: Are we ready to commit rapidly to such promises?

## Figures and Tables

**Table 1:** Key differences between a healthy (eubiosis) and an imbalanced (dysbiosis) lung microbiota, emphasizing their impact on lung health.

Feature	Eubiosis	Dysbiosis
**Definition**	A balanced and healthy state of microbiota	An imbalanced state of lung microbiota
**Microbial Composition**	Low levels and high diversity of beneficial microbes.	Increased levels and reduced diversity, of harmful microbes.
**Host-Microbe Interaction**	Symbiotic relationship. Supports lung health.	Disrupted interaction. Contributes to lung diseases.
**Microbial Metabolites**	Production of beneficial metabolites like short-chain fatty acids (SCFAs).	Accumulation of harmful metabolites that may exacerbate lung conditions.
**Immune Response**	Supports a balanced immune surveillance against pathogens and tumor cells.	Hyperactive immune response, leading to tissue damage.
**Inflammation Levels**	Low or controlled.	Elevated or chronic.
**Barrier Function**	Intact epithelial barrier function, protecting lung tissue.	Compromised barrier function, increasing susceptibility to infections.
**Environmental Impact**	Less affected by environmental factors like pollutants and smoking.	Highly influenced by external factors, worsening dysbiosis.
**Impact on Lung Health**	Support Healthy lung function, resistance to infections, and tumor suppression.	Associated with respiratory diseases like asthma, COPD, infections, and lung cancer.

## Data Availability

Not applicable.
